# Cochrane systematic reviews as a source of information for practice and trials

**DOI:** 10.1186/1745-6215-12-S1-A49

**Published:** 2011-12-13

**Authors:** Mike Clarke, Thomas T Clarke, Lorcan Clarke

**Affiliations:** 1 All-Ireland Hub for Trials Methodology Research, Queen’s University Belfast, Northern Ireland; 2 UK Cochrane Centre, National Institute for Health Research, Oxford, UK

## Background

Systematic reviews should be used to provide the ethical, scientific and environmental justification for a new randomised trial [1]. The Cochrane Collaboration is the world’s largest organisation dedicated to preparing and maintaining systematic reviews of the effects of healthcare interventions [2]. These are published in the *Cochrane Database of Systematic Reviews* (*CDSR*) and all Cochrane Reviews have a rigid structure, which includes the authors’ conclusions on the implications for practice. Therefore, this collection of reviews provides a readily available source of information on the evidence base for the use of a large number of interventions in health care.

## Objectives

We assessed all new and updated reviews in the first twelve monthly issues of *CDSR* from February 2010 to January 2011 to identify their relevance to the evidence base for current practice and for future trials to resolve continuing uncertainties.

## Methods

At least two authors independently examined the implications for practice in the authors’ conclusions of each new and updated review in issues 2-12 2010 and issue 1 2011 of *CDSR*. Each review was coded to indicate whether its authors concluded that a specific intervention should only be used in research, was not supported or refuted by the evidence in the review, had been shown to be effective, or should not be used in practice (or could not be recommended). Each review was also coded by area of health or health care. The final decisions on coding were taken by one author.

## Results

These 12 monthly issues of *CDSR* contained 390 new and 462 updated reviews, many of which provide evidence on more than one intervention. The authors of 25 (2.9%) of these reviews concluded that an intervention should only be used in research and 494 (58.0%) concluded that the evidence for an intervention was insufficient to support or refute its use. At least one intervention in 413 (48.5%) of these reviews was judged to be effective by the review authors and the authors concluded that an intervention should not be used or could not be recommended in 139 (16.3%) reviews.

## Conclusions

Cochrane Reviews identify many interventions for which the research evidence supports their effectiveness and many where the evidence is insufficient to assess benefits and harms. This latter group, along with reviews in which the authors recommend restricting an intervention to use in research, provide examples of uncertainties which could be resolved through randomised trials.
